# Snake optimizer LSTM-based UWB positioning method for unmanned crane

**DOI:** 10.1371/journal.pone.0293618

**Published:** 2023-11-01

**Authors:** Li Wang, Guangxiao Fan, Qiao Wang, Hui Li, Junhai Huo, Shibo Wei, Qunfeng Niu

**Affiliations:** 1 College of Electrical Engineering, Henan University of Technology, Zhengzhou City, Henan Province, China; 2 Key Laboratory of Grain Information Processing and Control (Henan University of Technology), Ministry of Education, Zhengzhou City, Henan Province, China; 3 Henan Key Laboratory of Grain Photoelectric Detection and Control, Henan University of Technology, Zhengzhou City, Henan Province, China; 4 College of Mechanical and Electrical Engineering, Henan University of Technology, Zhengzhou City, Henan Province, China; 5 Guizhou Aerospace Control Technology Limited Company, Guiyang City, Guizhou Province, China; TU Wien: Technische Universitat Wien, AUSTRIA

## Abstract

Position determination is a critical technical challenge to be addressed in the unmanned and intelligent advancement of crane systems. Traditional positioning techniques, such as those based on magnetic grating or encoders, are limited to measuring the positions of the main carriage and trolley. However, during crane operations, accurately determining the position of the load becomes problematic when it undergoes swinging motions. To overcome this limitation, this paper proposes a novel Ultra-Wide-Band (UWB) positioning method for unmanned crane systems, leveraging the Snake Optimizer Long Short-Term Memory (SO-LSTM) framework. The objective is to achieve real-time and precise localization of the crane hook. The proposed method establishes a multi-base station and multi-tag UWB positioning system using a Time Division Multiple Access (TDMA) combined with Two-Way Ranging (TWR) scheme. This system enables the acquisition of distance measurements between the mobile tag and UWB base stations. Furthermore, the hyperparameters of the LSTM network are optimized using the Snake Optimizer algorithm to enhance the accuracy and effectiveness of UWB positioning estimation. Experimental results demonstrate that the SO-LSTM-based positioning method yields a maximum positioning error of 0.1125 meters and a root mean square error of 0.0589 meters. In comparison to conventional approaches such as the least squares method (LS) and the Kalman filter method (KF), the proposed SO-LSTM-based positioning method significantly reduces the root mean square error (RMSE) by 63.39% and 58.01%, respectively, while also decreasing the maximum positioning error (MPE) by 60.77% and 52.65%.

## 1. Introduction

Within the framework of the "Made in China 2025" initiative, traditional cranes are undergoing a transformation towards intelligent and unmanned configurations. The realization of intelligent and unmanned crane systems necessitates the overcoming of key technological challenges, including positioning, decision-making planning, and tracking control [1, 2]. Notably, positioning serves as a fundamental requirement for enabling effective decision-making planning and tracking control. The encoder positioning method, which translates the rotational angle of the shaft into distance measurements fed into the control system, is a viable option for conventional cranes. However, its suitability diminishes when higher positioning accuracy is desired. This limitation stems from two primary factors. Firstly, occasional slight slippage of the crane’s active wheels leads to shaft rotation without corresponding displacement, thereby compromising the stability of the positioning system. Secondly, the variation in track height causes one of the wheels to lose contact with the track over short distances, a phenomenon significantly influenced by track quality [3]. Alternatively, the barcode positioning system relies on barcode locators and barcodes to facilitate measurement and positioning. This system scans barcode graphics installed along the crane’s running line and derives position information by comparing the scanned data with the stored information in memory. In contrast to the encoder positioning system, the barcode positioning system effectively mitigates the challenges associated with wheel slippage and substandard track quality. Nevertheless, neither the encoder positioning system nor the barcode positioning system can accurately determine the position of the crane hook during swinging motions [4].

The increasing demand for intelligent and unmanned operations in cranes necessitates a positioning system that can meet the advanced requirements. Traditional positioning methods such as encoder positioning and barcode positioning fall short in satisfying the positioning needs of unmanned driving cranes. Consequently, researchers have been actively exploring indoor positioning methods suitable for unmanned driving in crane applications. Notable approaches include Wi-Fi positioning, Bluetooth positioning, geomagnetic positioning, pedestrian dead reckoning, and UWB positioning methods [5]. While Wi-Fi positioning has gained popularity, its accuracy remains relatively low. Wi-Fi fingerprint-based positioning methods require extensive signal mapping prior to deployment, making them impractical for unmanned driving cranes [6–8]. Bluetooth positioning allows for short-range reception of information but suffers from significant distance estimation errors. Distance-based Bluetooth positioning methods fail to achieve high positioning accuracy, and the stability of Bluetooth signal strength poses challenges. Signal strength-based Bluetooth positioning also fails to deliver high accuracy [9, 10]. Magnetic positioning and pedestrian dead reckoning are often used in combination with other positioning methods due to the difficulty of achieving precise location estimates independently, resulting in limited positioning accuracy [11, 12]. Distance-based UWB indoor positioning leverages non-sinusoidal narrow-pulse transmission data, offering advantages such as fast transmission speed, low power consumption, high positioning accuracy, and robust anti-interference capabilities [13]. The study referenced as [14] conducts an analysis of the performance of linearized least squares estimation (LLSE), fingerprint estimation (FPE), and weighted centroid estimation (WCE) concerning their positioning accuracy within the context of UWB systems. The findings of this analysis serve to provide empirical evidence supporting the efficacy of UWB-based positioning methodologies.

Distance-based UWB positioning methods commonly employed include the Least Squares (LS) [15–17] and the Kalman Filtering (KF) [18–20]. In the context of indoor three-dimensional spatial positioning, LS method is susceptible to the influence of anchor geometric configurations, leading to challenges in effectively constraining the vertical dimension and subsequently resulting in elevated errors along the z-direction. Contrasted with the LS method, achieving heightened precision in position estimation through KF approach necessitates a more refined system model. Nonetheless, it is important to note that the KF method does not fundamentally resolve the influence of anchor geometric configurations on UWB positioning. In the literature, reference [21] extensively deliberates on the effect of anchor quantity on UWB position estimation accuracy, underscoring the incremental enhancement in UWB positioning precision as the number of anchors increases. Nonetheless, the augmented anchor count, while bolstering position estimation accuracy, concurrently entails escalated localization costs. Considering the rapid advancement of machine learning and deep learning techniques, the integration of artificial intelligence (AI) with positioning, commonly referred to as AI+ positioning, emerges as a promising alternative solution. The LSTM network is capable of extracting deep and abstract features from extensive sample data. It exhibits significant advantages in training and refining sequential data, enabling accurate position estimation with high precision without reliance on initial states.

In the training phase of LSTM, several factors influence the training outcomes, such as the sample size, data normalization technique, and hyperparameters of the LSTM network. Among these factors, the hyperparameters of the LSTM network play a critical role in determining the training results [22, 23]. Presently, researchers typically rely on their own expertise to determine the initial set of hyperparameters for LSTM networks and subsequently make iterative adjustments throughout the experimental process. However, this manual approach significantly impacts efficiency and frequently fails to yield the optimal parameter configuration.

Metaheuristic algorithms, encompassing genetic evolution-based algorithms, physics-based algorithms, and population-based algorithms, have gained considerable attention from engineers and researchers due to their simplicity, gradient-free nature, and powerful search capabilities [24]. In the domain of crane optimization, reference [25] introduced an enhanced seagull optimization algorithm, which outperformed other algorithms when applied to the optimization problem of the crane’s main beam. Moreover, reference [26] tackled the comprehensive scheduling problem involving dockside cranes and trucks by proposing an adaptive particle swarm optimization algorithm that yielded optimal solutions derived from mixed-integer programming. Additionally, reference [27] contributed an improved genetic algorithm specifically tailored for dockside crane scheduling, resulting in significantly reduced computational time while maintaining superior algorithmic performance compared to existing methods. The Snake Optimizer algorithm, introduced by Fatma A. Hashim in 2022, is a nature-inspired optimization technique that emulates the feeding and mating behavior of snakes to tackle diverse optimization tasks [28]. In this study, a novel approach is presented, namely the Snake Optimizer-based LSTM network, for UWB positioning in unmanned crane operations. The proposed method leverages the Snake Optimizer algorithm to optimize the hyperparameters of the LSTM network and achieve UWB localization. Through extensive experimental evaluations, it is demonstrated that the proposed SO-LSTM-based UWB localization algorithm enhances the positioning accuracy of existing crane systems, thus laying a solid foundation for the intelligence and unmanned aspects of crane operations. The primary contributions of this research are outlined as follows:

1. Constructed a multi-base station, multi-tag UWB positioning system, collected UWB distance measurements and Tag positions in a real crane scenario, built a UWB ranging dataset for crane applications, and applied it to determine the position of lifted objects by cranes.
2. This paper proposes the use of the Snake Optimizer algorithm to solve the problem of optimizing the hyperparameters of the LSTM network, and verifies the feasibility of using the Snake Optimizer algorithm to find the optimal hyperparameters of the LSTM network.
3. The application of the SO-LSTM-based UWB positioning algorithm in the UWB positioning system for cranes enables the determination of the position of the crane’s hook when the lifted object swings. This offers a novel positioning method solution for unmanned crane systems.

The subsequent sections of this manuscript are structured as follows: Chapter 2 elaborates on the application of deep learning techniques in UWB positioning. Chapter 3 provides an in-depth discussion on the network model employed in the multi-base station and multi-label crane UWB positioning system. Chapter 4 elucidates the proposed UWB positioning method based on the SO-LSTM framework. In Chapter 5, the efficacy of the positioning algorithm is rigorously examined and validated through a series of comprehensive experiments. Lastly, Chapter 6 presents a concise summary of the accomplishments and contributions made in this research endeavor.

## 2. Related work

Scholars both domestically and internationally have conducted extensive research on UWB positioning. Reference [29], proposed as early as 2012 the utilization of UWB for estimating the posture of cranes, a crucial aspect for predicting potential collisions involving cranes. As research endeavors continue to delve deeper, recent years have witnessed new breakthroughs in UWB technology. In this chapter, we delve into the advantages of deep learning networks and their application within UWB positioning systems.

In recent years, deep learning has received considerable attention in the field of positioning. In reference to [30], the CNN-LSTM deep learning method was employed for UWB NLOS/LOS signal classification, demonstrating superior classification performance. Another study presented in reference [31] proposed a novel positioning method that leverages convolutional neural networks (CNNs) in conjunction with UWB signals. Through simulations, it was verified that the proposed CNN-based positioning method outperforms traditional threshold-based approaches. In the context of mitigating range errors in NLOS conditions, reference [32] introduced a deep learning-based approach that exploits the distinct response states exhibited by the channel impulse response (CIR). Experimental evaluations conducted in a greenhouse with multiple intervals demonstrated significant mitigation of positioning errors in NLOS environments, while retaining usability even in severely obstructed scenarios. To address the challenges posed by multipath and non-line-of-sight propagation in UWB signals, reference [33] proposed a novel positioning framework. By extracting features from time and power vectors obtained from UWB signals, a multi-layer perceptron (MLP) and a convolutional neural network (CNN) were employed to enhance the performance of indoor positioning systems. Simulation results indicated that the designed CNN architecture reduced the average error by approximately 3 cm. In the realm of gated recurrent unit (GRU)-based positioning methods, reference [34] introduced a positioning approach that combines GRU with UWB signals. Compared to existing methods based on CNN, this approach significantly reduces training and testing time. Simulation results demonstrated that the proposed method achieved a low RMSE of 0.8 meters. Furthermore, reference [35] proposed a deep learning model utilizing LSTM networks for UWB tag position prediction. The performance of the proposed LSTM-based UWB positioning system was analyzed in terms of learning rate, loss function, and optimizer selection. Simulation results showcased that the proposed UWB positioning solution achieved centimeter-level average positioning error, surpassing traditional UWB positioning methods.

In the "Related Work" section, discussions revolve around the utilization of machine learning concepts to enhance the accuracy of UWB system positioning. However, the positioning accuracy of UWB systems still requires further enhancement. Therefore, it is imperative to introduce a novel positioning methodology tailored for the unmanned crane’s location estimation. With the objective of minimizing positioning errors, this paper proposes a novel UWB positioning approach for unmanned cranes based on the SO-LSTM framework. Experimental results demonstrate that, in comparison to traditional LS and KF methods, this approach effectively reduces the UWB system’s positioning errors. This contribution holds significant implications for the future development of unmanned autonomous crane systems.

## 3. Multi-anchor multi-tag UWB localization system network model

The UWB positioning system comprises three main components: the Tag, the Anchor, and the Central Localization Engine (CLE) residing in the backend. The CLE is responsible for clock synchronization with the Anchors. Upon receiving a beacon request from an Anchor, the CLE initializes the data to ensure consistency with the Anchors’ data within the system. Throughout the system’s operation, the CLE continuously monitors the status of both the Anchors and Tags. Collaboratively, the Tag and Anchor perform distance measurements, which are integral to the functionality of the UWB positioning system.

**Fig 1:**
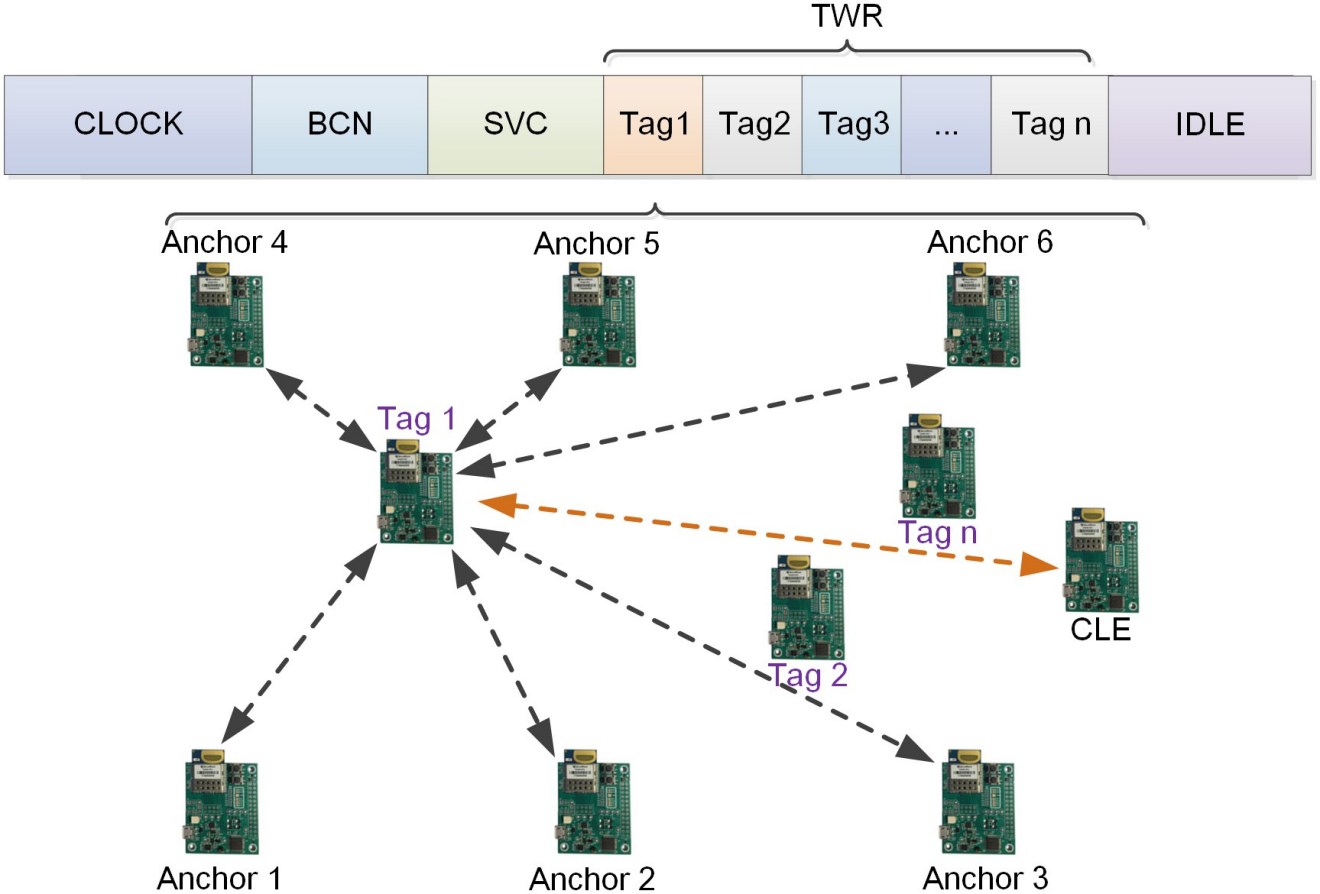
Network model and time slot allocation.

The UWB positioning system consists of a set of n tags, employing TDMA technology to partition time into various time slots for specific purposes such as clock synchronization, base station broadcasting, service channel (SVC) reservation, TWR distance measurement, and IDLE protection time. The CLOCK time slot is designated for clock synchronization between the CLE and the Anchors within the system. The BCN time slot is utilized by Anchors to request time slots. The network model and slot allocation of the multi-anchor multi-tag UWB positioning system are depicted in Fig 1 Each Tag is assigned a dedicated time slot, wherein *Tag* 1, for example, operates while other Tags remain in standby mode. *Tag* 1 employs the TWR distance measurement principle to calculate the distance between itself and the Anchors within the system.

**Fig 2:**
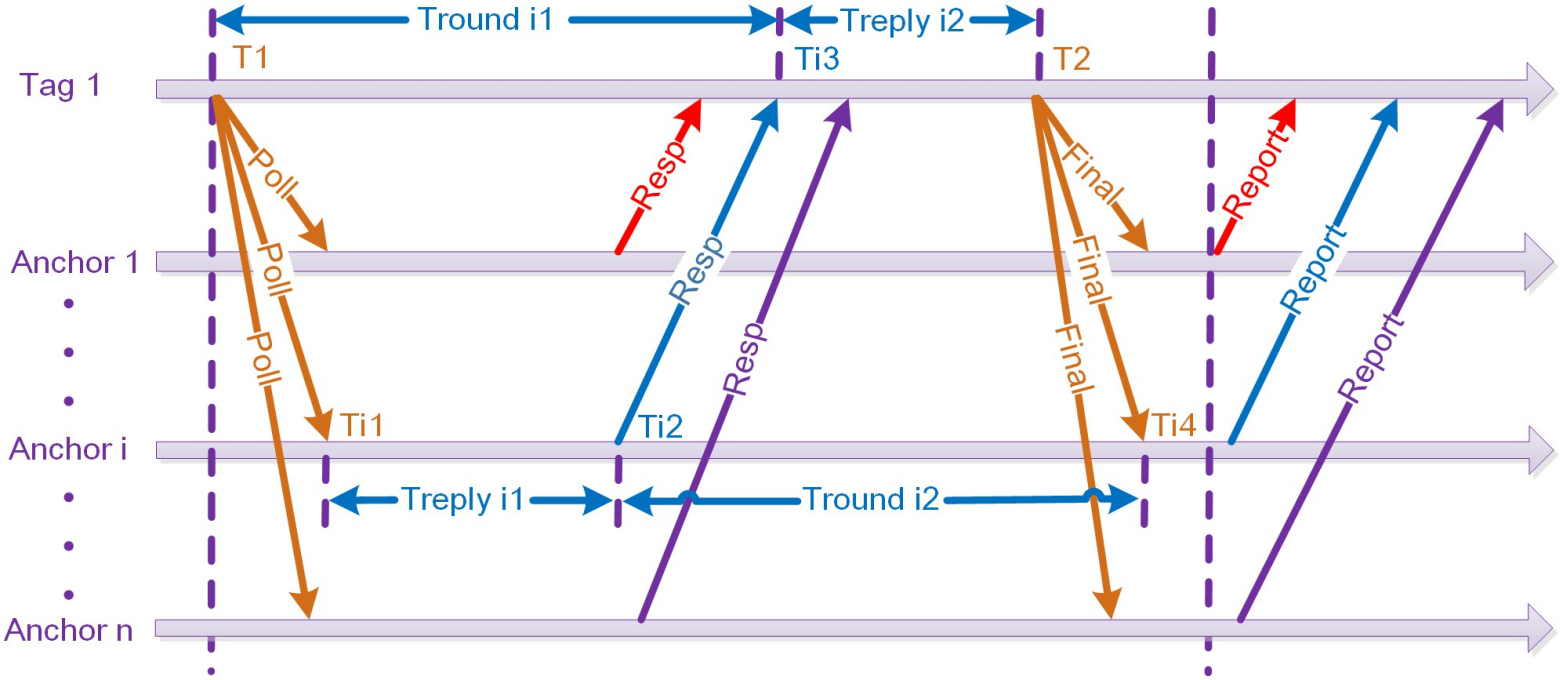
Time of flight ranging model.

The TWR distance measurement technique estimates the distance between the Tag and Anchor by measuring the round-trip flight time of the signal transmitted between them. Fig 2 illustrates the distance measurement model employed between the Tag and Anchor. The process begins with the Tag initiating a distance measurement request called "Poll" and noting the transmission time *T*1. Subsequently, the i-th Anchor receives the "Poll" request from the Tag and records the arrival time *Ti*1. *Anchor i* responds to the Tag by sending a response message, denoted as "Resp," while also recording the transmission time *Ti*2. The Tag receives the response message from *Anchor i*, noting the reception time *Ti*3. Finally, the Tag transmits a final ranging message called "Final," recording the transmission time *T*2. *Anchor i* receives the ranging message and records the reception time *Ti*4.

*Anchor i* derives the round-trip timestamp measurements between the Tag and Anchor, resulting in the values *T*_*roundi*1_, *T*_*roundi*2_, *T*_*replyi*1_, and *T*_*replyi*2_. *T*_*round*_ signifies the time interval encompassing the transmission of a ranging pulse signal by the Tag or Anchor to the subsequent reception of the ranging pulse signal. *T*_*reply*_ denotes the time interval spanning the reception of a ranging pulse signal by the Tag or Anchor to the subsequent transmission of the ranging pulse signal. The computation of the flight time is performed as follows:

$$T_{prop}=\frac{T_{roundi1}\times T_{roundi2}-T_{replyi1}\times T_{replyi2}}{T_{roundi1}+T_{roundi2}+T_{replyi1}+T_{replyi2}}$$ (1)

Upon completion of the distance measurement process, *Anchor i* transmits a Report message to the Tag, conveying the distance information between *Anchor i* and the Tag. The Tag then encapsulates this information and uniformly transmits it to the CLE for further processing. The CLE initially employs the LS method to preliminarily compute the position of the Tag. Subsequently, the distance information, along with the computed Tag position, is transmitted to the computer, where further refinement of the position estimation is carried out.

## 4. The UWB positioning method for unmanned tower cranes based on SO-LSTM

The Snake Optimizer algorithm, inspired by the feeding and mating behavior of snakes, is a nature-inspired optimization algorithm employed in this study. The proposed UWB positioning method based on SO-LSTM consists of two stages. In the initial stage, the Snake Optimizer algorithm is utilized to optimize the hyperparameters of the LSTM network. Subsequently, in the second stage, the LSTM network is trained to accurately predict the position of the tag.

As depicted in Table 1, the distance measurement values obtained from the UWB positioning system in this study are categorized into two groups: the optimization group and the experimental group. The optimization group is employed for optimizing the hyperparameters of the LSTM network using the Snake Optimizer algorithm. On the other hand, the experimental group is utilized for training the LSTM network and predicting the position of the UWB Tag. Both groups encompass training data and testing data, which comprise distance measurement values and the corresponding Tag positions obtained from measurements.

**Table 1:** UWB data grouping.

UWB Data			
Optimization Group		Experimental Group	
training data	test data	training data	test data

### 4.1. Snake optimizer LSTM hyperparameters

The Snake Optimizer algorithm is employed to perform hyperparameter optimization for the LSTM network, aiming to identify improved training parameters suitable for UWB positioning. The hyperparameters considered in this study for optimization using the Snake Optimizer algorithm encompass the number of hidden nodes, learning rate, and number of iterations of the LSTM network. These hyperparameters play a crucial role in enhancing the performance and effectiveness of the LSTM network for UWB positioning.

**Fig 3:**
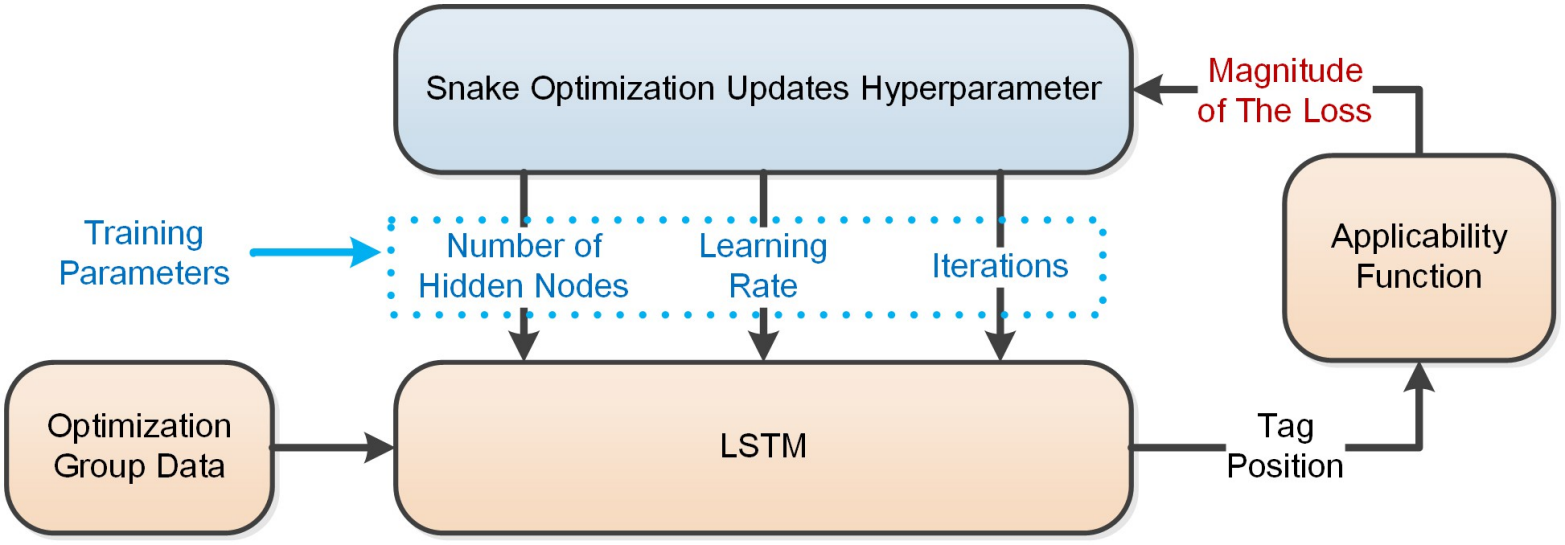
Snake optimizer algorithm optimization process.

Illustrated in Fig 3, the optimization of LSTM network hyperparameters via the Snake Optimizer algorithm involves a iterative process. The algorithm continuously updates the training parameters and trains the LSTM network using the generated hyperparameters to estimate the position of the UWB Tag. The fitness function is responsible for evaluating the loss value of the LSTM network. The Snake Optimizer algorithm receives the loss value computed by the fitness function, generates refined training parameters, and subsequently feeds them back to the LSTM network for further training. The performance of the trained parameters is considered better when the loss value obtained from the fitness function is smaller. The calculation method employed by the fitness function is outlined as follows:

$$rmse=sqrt(\frac{1}{n}\times \sum_{i=1}^{n}\left(\Delta x_{i}{}^{2}+\Delta y_{i}{}^{2}+\Delta z_{i}{}^{2}\right))$$ (2)

In this context, Δ*x*_*i*_, Δ*y*_*i*_, and Δ*z*_*i*_ denote the discrepancies between the estimated position and the measured position of the i-th distance measurement value along the x-axis, y-axis, and z-axis, respectively.

**Fig 4:**
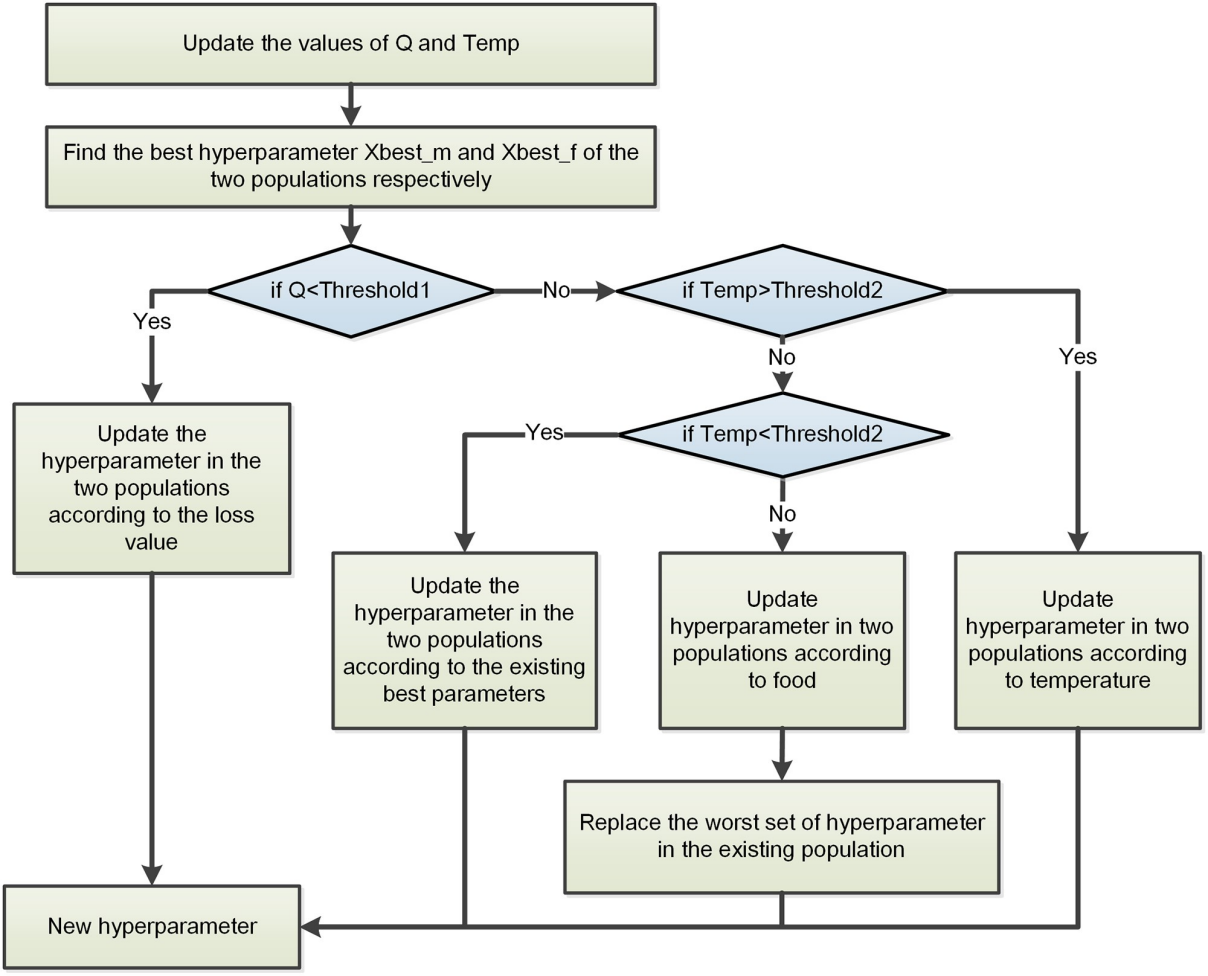
The process of updating LSTM hyperparameters with the snake optimizer algorithm.

Initially, the Snake Optimizer algorithm randomly generates n sets of parameters and proceeds to train the LSTM network using each parameter set, while recording the corresponding training loss. The algorithm then identifies the parameter set that yields the smallest loss as the global best parameter, denoted as *X*_*food*_. Subsequently, the n parameter sets are evenly divided into two groups: the male parameter group and the female parameter group. The best parameters *f*_*best*,*m*_ for the male group and *f*_*best*,*f*_ for the female group are determined. The relevant parameters associated with the Snake Optimizer algorithm are presented in Table 2. The process of updating the LSTM network hyperparameters using the Snake Optimizer algorithm is depicted in Fig 4.

**Table 2:** SO-LSTM algorithm parameters.

Symbol	Value	Parameter
Threshold1	0.25	Food quantity (Q) threshold
Threshold2	0.6	Environment temperature (Temp) threshold
[X_1min_, X_1max_]	[10, 200]	Search Range for Hidden Node Count
[X_2min_, X_2max_]	[0.001, 0.02]	Search Range for Learning Rate
[X_3min_, X_3max_]	[10, 500]	Search Range for Maximum Iterations

During the optimization process, the maximum number of optimization iterations is represented as T, while the current iteration is denoted as t. Initially, the quantities of food (Q) and environmental temperature (Temp) are computed, and their values are influenced by T and t. The calculation formulas for Q and Temp are as follows:

$$Temp=\text{exp}(\frac{-t}{T})$$ (3)

$$Q=C_{1}*\text{exp}(\frac{t-T}{T})$$ (4)

During the optimization process, various behaviors are performed based on the comparison between the Q and the predefined threshold value (Threshold1). If Q is less than Threshold1, the new parameters are determined by updating the current parameters solely based on the loss value. The formulas for updating the male parameter group are as follows:

$$A_{m}=\text{exp}(\frac{-f_{rand,m}}{f_{i,m}})$$ (5)

$$X_{i,m}(t+1)=X_{rand,m}(t)\pm C_{2}\times A_{m}\times ((X_{\text{max}}-X_{\text{min}})\times rand+X_{\text{min}})$$ (6)

Formula for calculating the female parameter group:

$$A_{f}=\text{exp}(\frac{-f_{rand,f}}{f_{i,f}})$$ (7)

$$X_{i,f}=X_{rand,m}(t+1)\pm C_{2}\times A_{f}\times ((X_{\text{max}}-X_{\text{min}})\times rand+X_{\text{min}})$$ (8)

*X*_*i*_ refers to the position of the i-th parameter in the parameter group, *m* represents the male parameter group, *f* represents the female parameter group, and *rand* is a random number between 0 and 1.

If the food amount Q is greater than the threshold value Threshold1, the algorithm further examines whether the environmental temperature Temp exceeds the threshold value Threshold2. In the case where Temp is greater than Threshold2, both the male and female parameter groups are updated based on the environment temperature.


$$X_{i,j}(t+1)=X_{food}\pm C_{3}\times Temp\times rand\times (X_{food}-X_{i,j}(t))$$ (9)

If the food amount Q is greater than the threshold value Threshold1 and the environmental temperature Temp is less than the threshold value Threshold2, the parameter group is updated based on the current best parameters. The calculation formula for updating the male parameter group is as follows:

$$FM=\text{exp}(\frac{-f_{best,f}}{f_{i}})$$ (10)

$$X_{i,m}(t+1)=X_{i,m}(t)+C_{3}\times FM\times rand\times (Q\times X_{best,f}-X_{i,m}(t))$$ (11)

Calculation formula for the female parameter group:

$$X_{i,f}(t+1)=X_{i,f}(t+1)+C_{3}\times FF\times rand\times (Q\times X_{best,m}-X_{i,f}(t+1))$$ (12)

If the food amount Q is greater than the threshold value Threshold1 and the environmental temperature Temp is equal to the threshold value Threshold2, the parameter group is updated based on the food quantity, and the worst-performing group in the existing parameter group is replaced. The calculation formula for updating the parameter group according to the food quantity is as follows: The calculation formula for updating the male parameter group is:

$$X_{i,m}(t+1)=X_{i,m}(t)+C_{3}\times M_{m}\times rand\times (Q\times X_{i,f}(t)-X_{i,m}(t))$$ (13)

The newly generated set of parameters replaces the worst-performing set of parameters in the male parameter group.


$$X_{worst,m}=X_{\text{min}}+rand\times (X_{\text{max}}-X_{\text{min}})$$ (14)

The calculation formula for updating the female parameter group is as follows: the newly generated set of parameters replaces the worst-performing set of parameters in the female parameter group.


$$X_{i,f}(t+1)=X_{i,f}(t)+C_{3}\times M_{f}\times rand\times (Q\times X_{i,m}(t)-X_{i,f}(t))$$ (15)

The worst-performing set of parameters in the male parameter group is replaced with the newly generated set of parameters.


$$X_{worst,f}=X_{\text{min}}+rand\times (X_{\text{max}}-X_{\text{min}})$$ (16)

The Snake Optimizer algorithm updates the parameters and conducts training of the LSTM network using the generated parameters. It outputs the training loss of the network with these parameters and updates the parameters based on the obtained loss value. Upon completion of the optimization process, the algorithm examines whether the current optimization count t is less than the maximum optimization count T. If t is indeed less than T, the algorithm proceeds with further optimization; otherwise, it outputs the best set of hyperparameters obtained.

### 4.2. LSTM localization method

**Fig 5:**
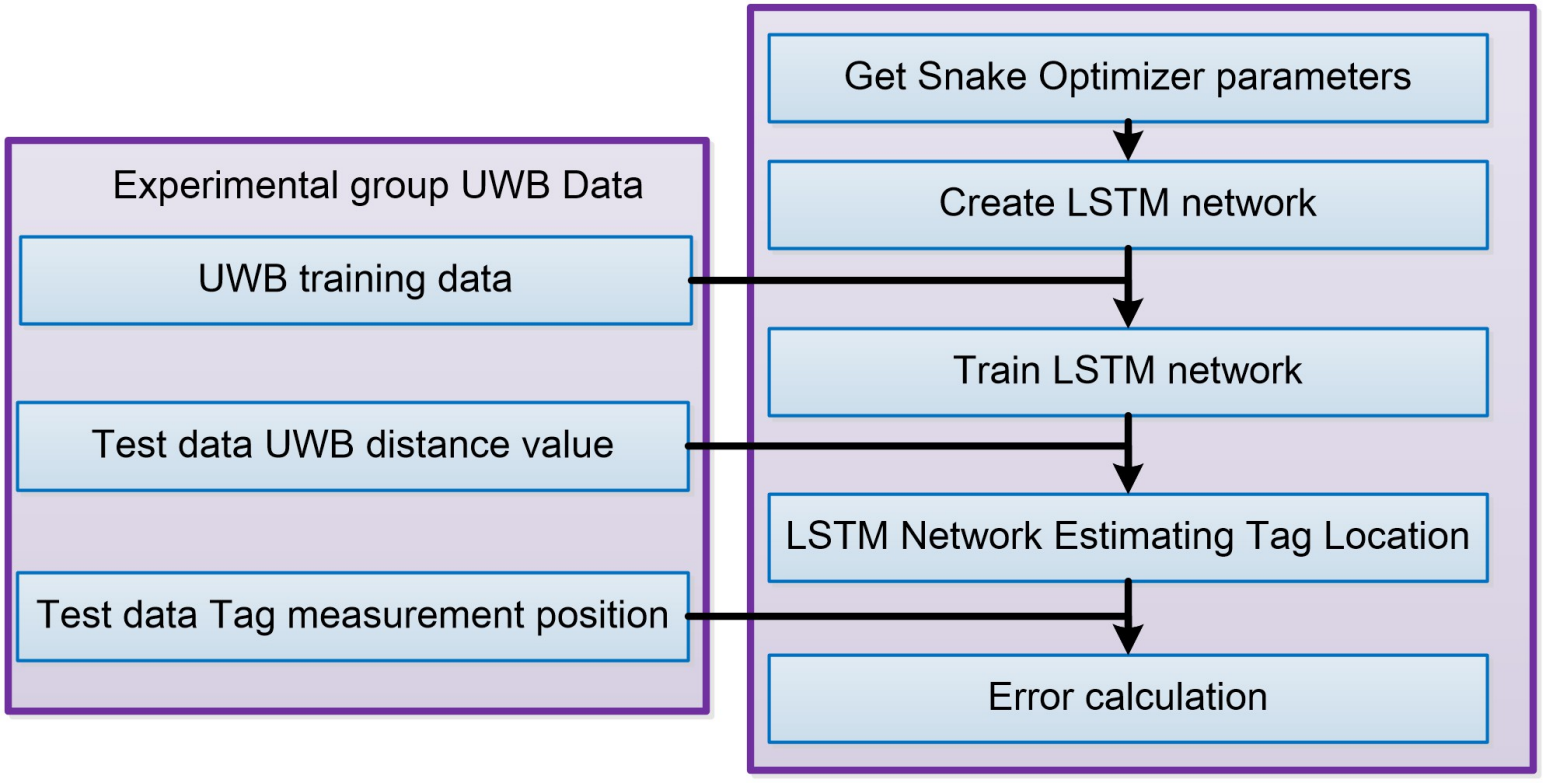
Process of UWB location prediction based on LSTM.

The UWB ranging data exhibits temporal correlation, and considering the comparison with Recurrent Neural Network (RNN) and Extreme Learning Machine (ELM) models, the LSTM model is determined to be more suitable for UWB localization. Accordingly, this paper implements a UWB positioning prediction process based on LSTM, as depicted in Fig 5.

1. The LSTM network hyperparameters optimized by the Snake Optimizer algorithm are obtained.
2. An LSTM network is constructed using the hyperparameters optimized by the Snake Optimizer algorithm, and the Adam optimization algorithm is employed to optimize and update the network’s parameters.
3. UWB training data for the experimental group is acquired, and the LSTM network created in Step (2) is trained using this data.
4. UWB ranging test data for the experimental group is obtained, and the trained LSTM network is utilized to predict the location of the UWB tag.
5. The predicted location generated by the LSTM network is analyzed, and a comparison is made with the measured location of the test data Tag in the experimental group. Subsequently, the RMSE and the MPE are calculated.

## 5. Experimental results and analysis

In order to assess the effectiveness of the proposed SO-LSTM-based UWB position estimation method, two sets of experiments were devised, wherein the position of the Tag was estimated using the LS, KF, and SO-LSTM methods respectively. In order to compare the positioning accuracy of these three methods, the study evaluated two performance metrics for each positioning method: RMSE and MPE.

### 5.1. Experiment platform introduction

**Fig 6:**
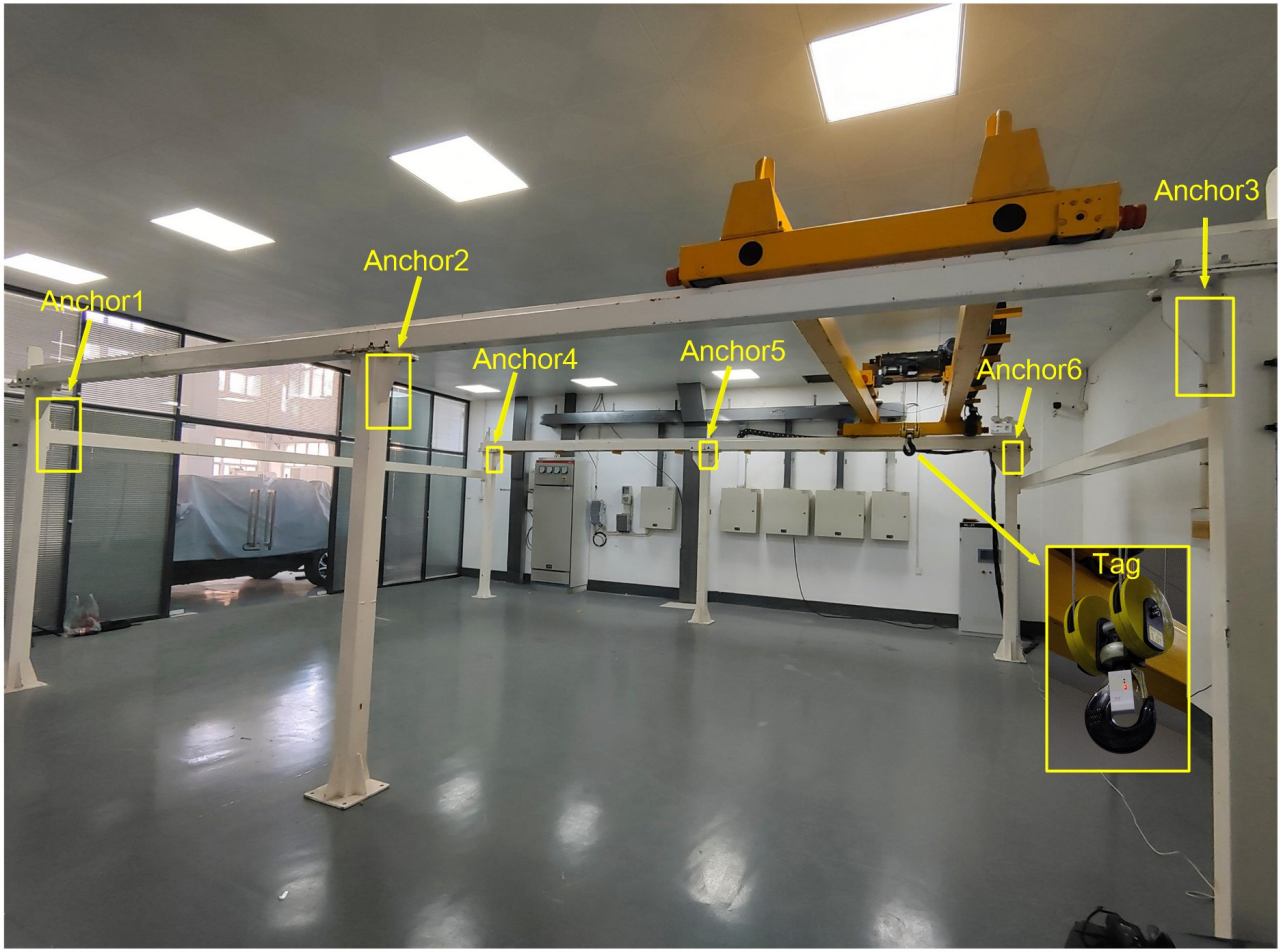
Crane experimental platform.

The experimental setup included an overhead bridge crane, as illustrated in Fig 6. The bridge crane had a total length of 7.8 meters and a total width of 5.8 meters. The main trolley of the crane had a maximum travel distance of 5.2 meters, while the auxiliary trolley had a maximum travel distance of 4 meters. The crane was capable of lifting loads weighing up to 1000 kilograms.

The experimental setup employed DWM1001 modules manufactured by Qorvo Semiconductor as the UWB devices. During the experimental setup, the UWB Anchors were positioned atop the support beam of the crane, while the UWB Tag was securely attached to the crane’s hook. A UWB ranging system consisting of 6 Anchors and 1 Tag was established for the experiment. The coordinates of the 6 Anchors were defined as follows: Anchor 1 (6.23, 0.01, 2.12), Anchor 2 (6.23, 3.68, 2.11), Anchor 3 (6.22, 7.35, 2.13), Anchor 4 (0.64, 0.03, 2.13), Anchor 5 (0.65, 3.7, 2.11), and Anchor 6 (0.64, 7.4, 2.11).

### 5.2. Experiment 1

**Fig 7:**
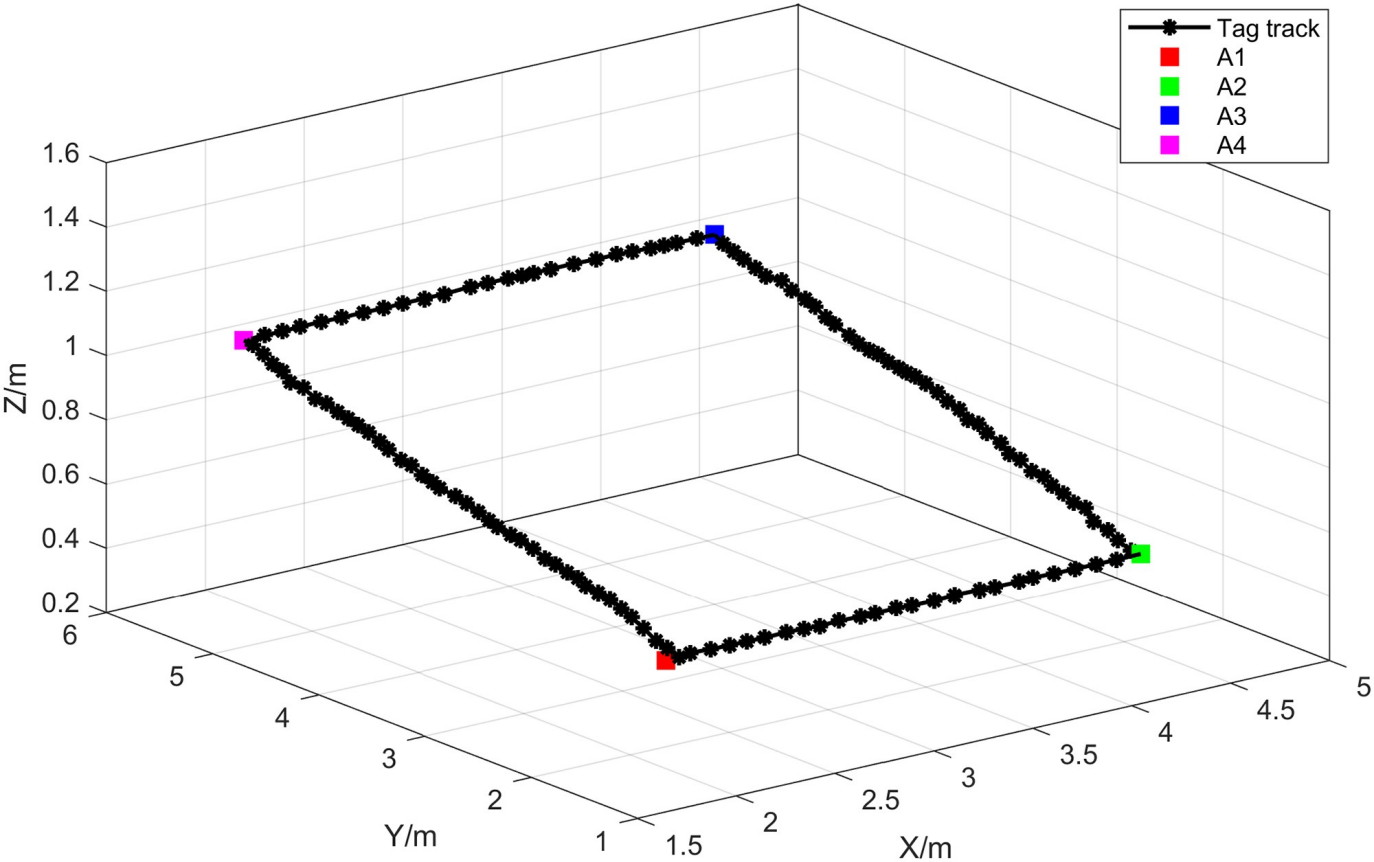
Experimental trajectory of crane hook movement in experiment 1.

**Fig 8:**
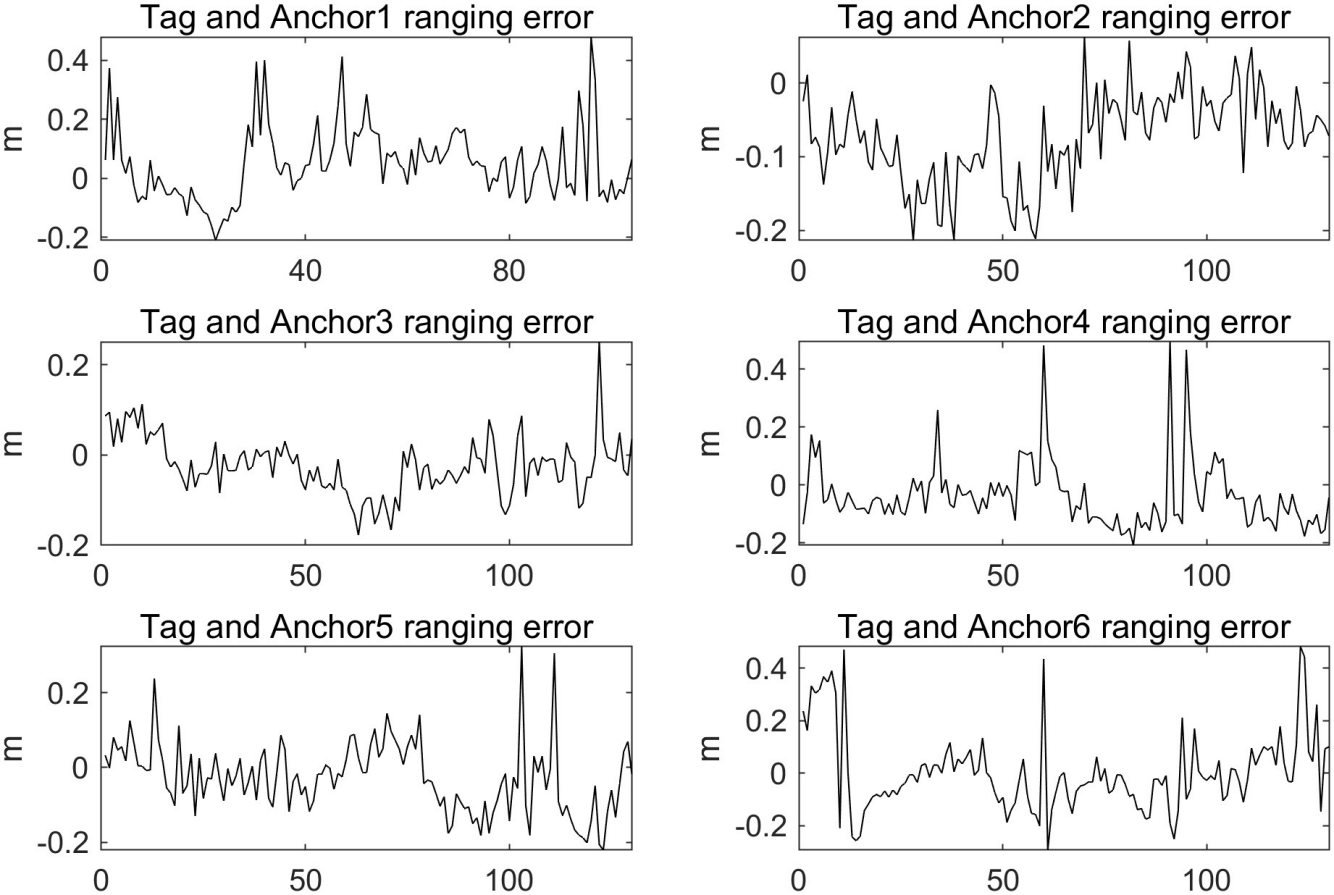
Visualization of ranging error in experiment 1.

In the first experiment, the initial position of the Tag was set at point A1 (2.006, 1.674, 0.535), with subsequent turning points at A2 (4.391, 1.642, 0.535), A3 (4.401, 5.673, 1.010), and A4 (2.041, 5.713, 1.008). The crane hook followed the trajectory A1→A2→A3→A4→A1. Throughout the crane’s movement, distance measurements between the Tag and the 6 Anchors were collected. The trajectory of the Tag’s movement is depicted in Fig 7. Calculate the errors between UWB distance measurements and actual distance values, and visualize the ranging errors. The visualized ranging errors are shown in Fig 8.

**Fig 9:**
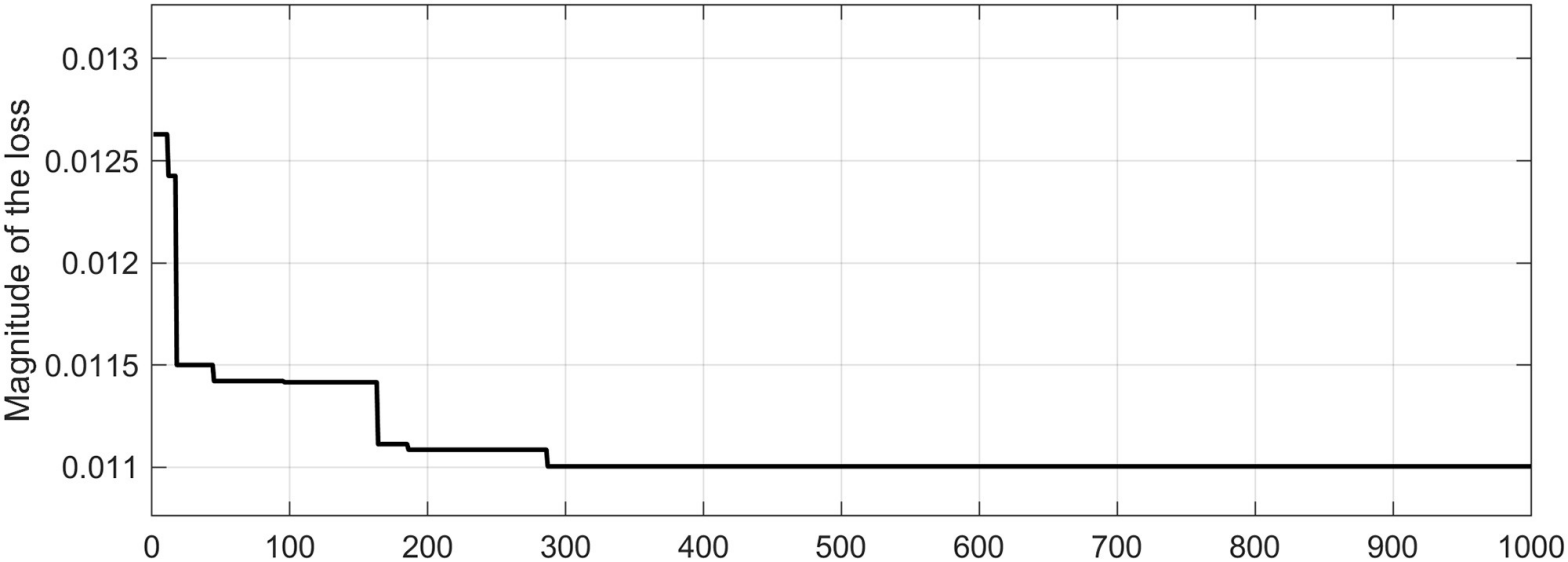
SO-LSTM loss value curve.

The Snake Optimizer algorithm iteratively generates new hyperparameters for the LSTM network and trains the network using these newly generated parameters. The fitness function evaluates the training loss, where a smaller loss indicates superior parameters. Through 1000 optimization iterations using the Snake Optimizer algorithm, the best hyperparameters for the LSTM network were determined as follows: 195 hidden nodes, a learning rate of 0.0182, and a maximum iteration count of 326. The loss value curve of the LSTM network during the Snake Optimizer algorithm optimization process is illustrated in Fig 9. The figure demonstrates a rapid convergence of the loss value, indicating the strong capability of the Snake Optimizer algorithm in optimizing the hyperparameters of the LSTM network.

**Fig 10:**
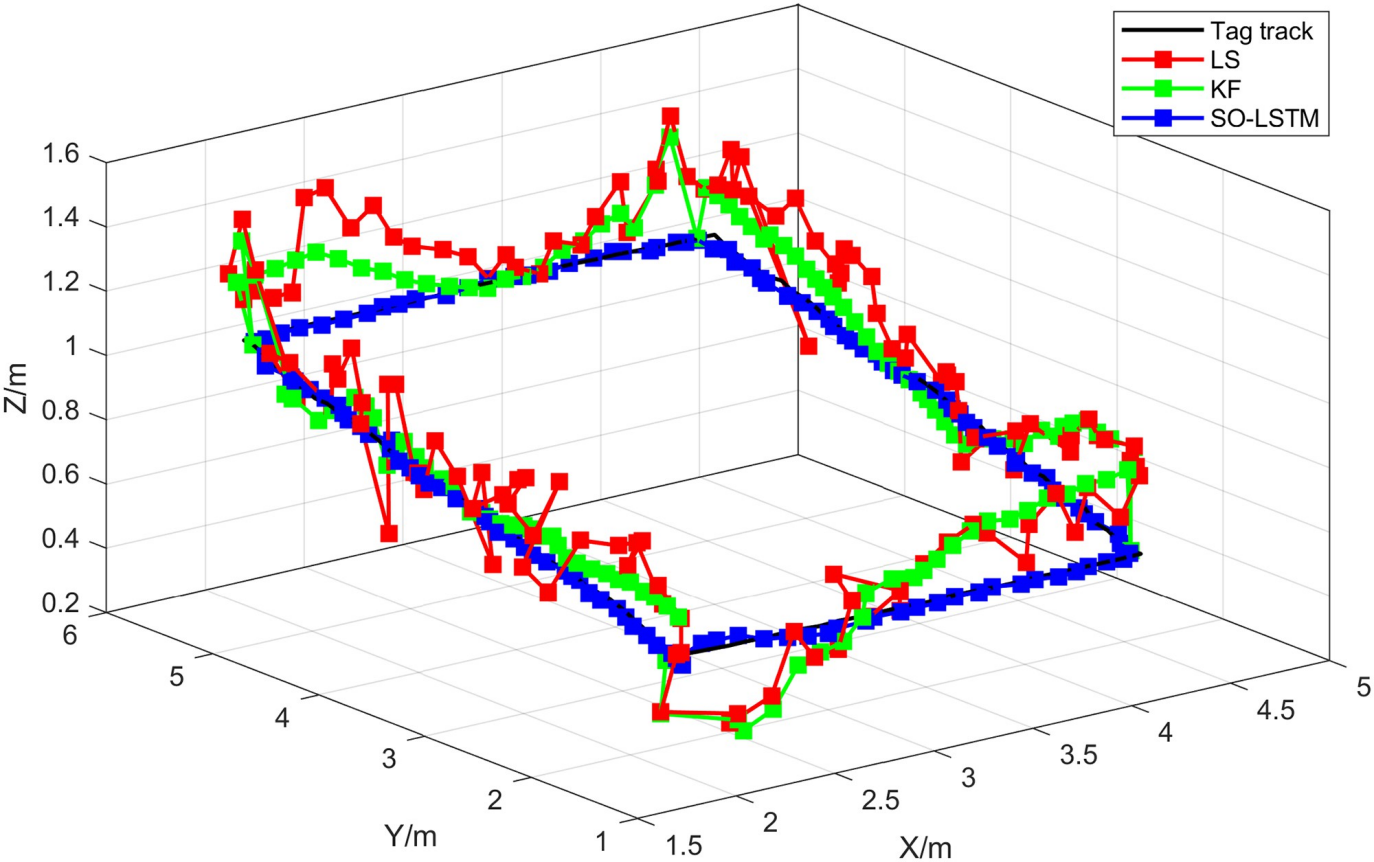
Localization trajectory for experiment 1.

Fig 10 presents the actual trajectory of the target alongside the estimated trajectories obtained using the LS, KF, and SO-LSTM localization methods. The evaluation of these localization methods in this experiment involved calculating the RMSE and MPE. As shown in Table 3. For the LS method, the calculated RMSE and MPE were 0.1999 meters and 0.3354 meters, respectively. The KF method yielded an RMSE of 0.1510 meters and an MPE of 0.2602 meters. As for the SO-LSTM localization method, the RMSE and MPE were found to be 0.0513 meters and 0.0917 meters, respectively. It is evident that the LS and KF methods exhibit larger fluctuations in target tracking, whereas the SO-LSTM localization method demonstrates reduced fluctuations and a stronger capability for tracking the target accurately.

**Table 3:** Statistical analysis of positioning errors for three localization methods in experiment 1.

	LS	KF	SO-LSTM
RMSE (m)	0.1999	0.1510	0.0513
MPE (m)	0.3354	0.2602	0.0917

**Fig 11:**
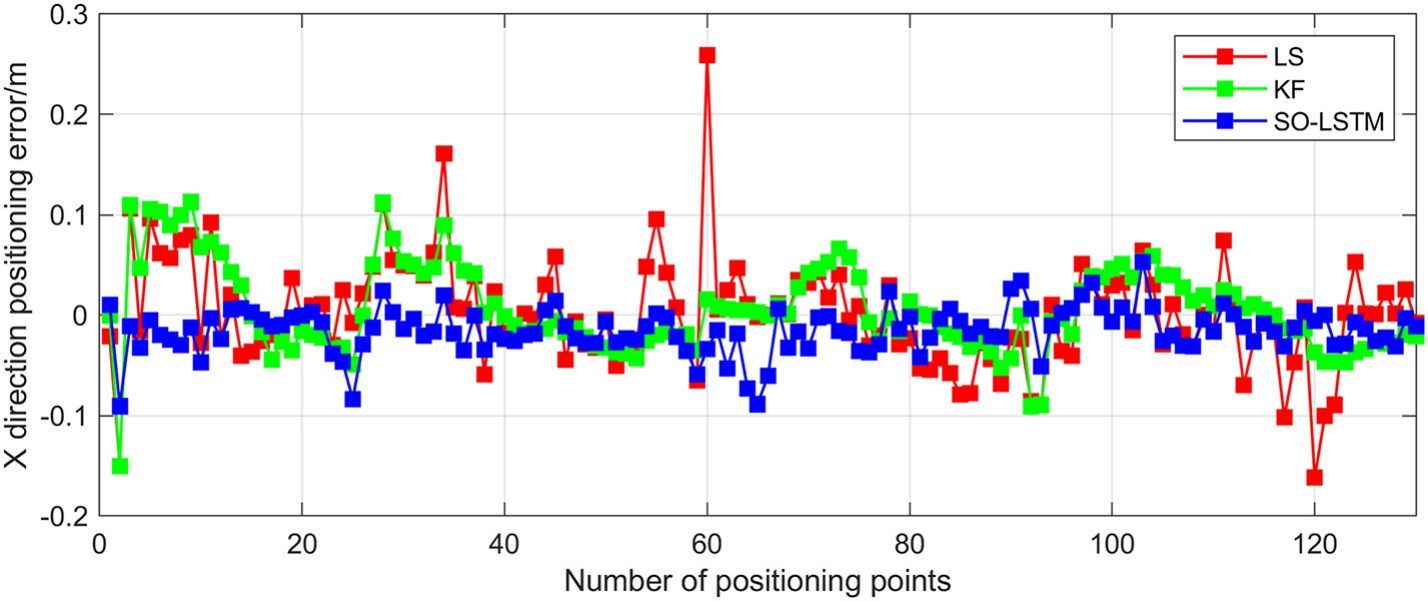
Experiment 1: X-axis positioning estimation error.

**Fig 12:**
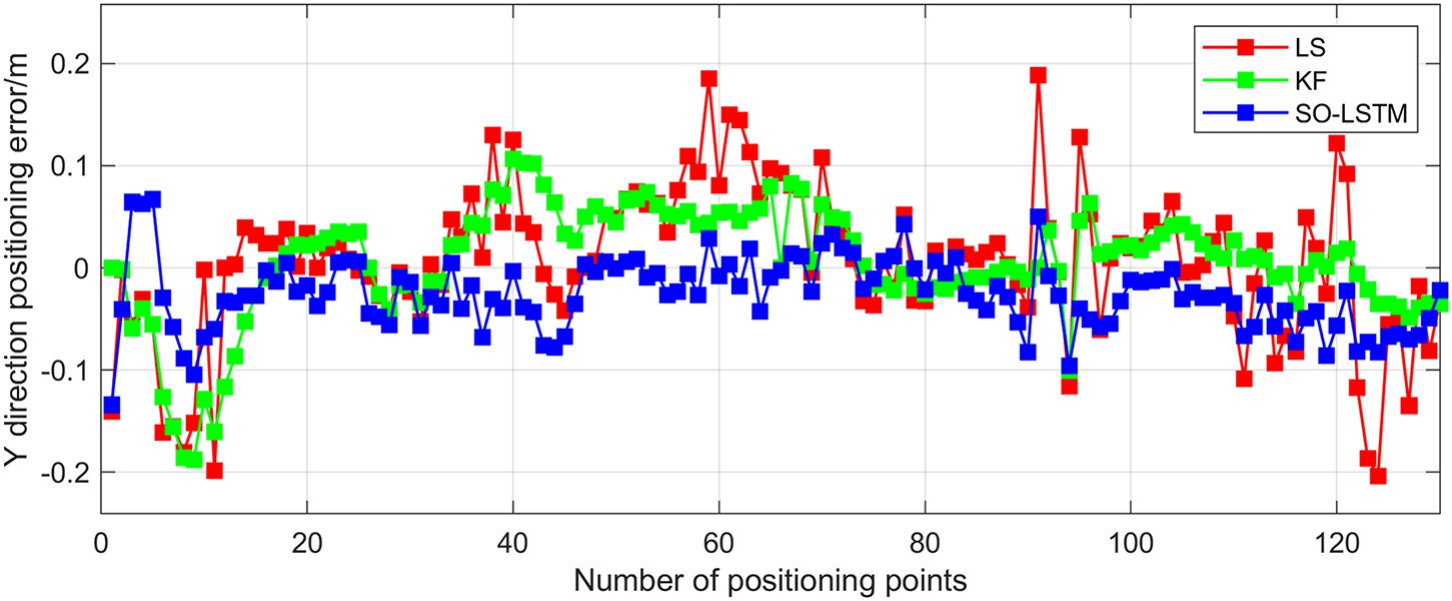
Experiment 1: Y-axis positioning estimation error.

**Fig 13:**
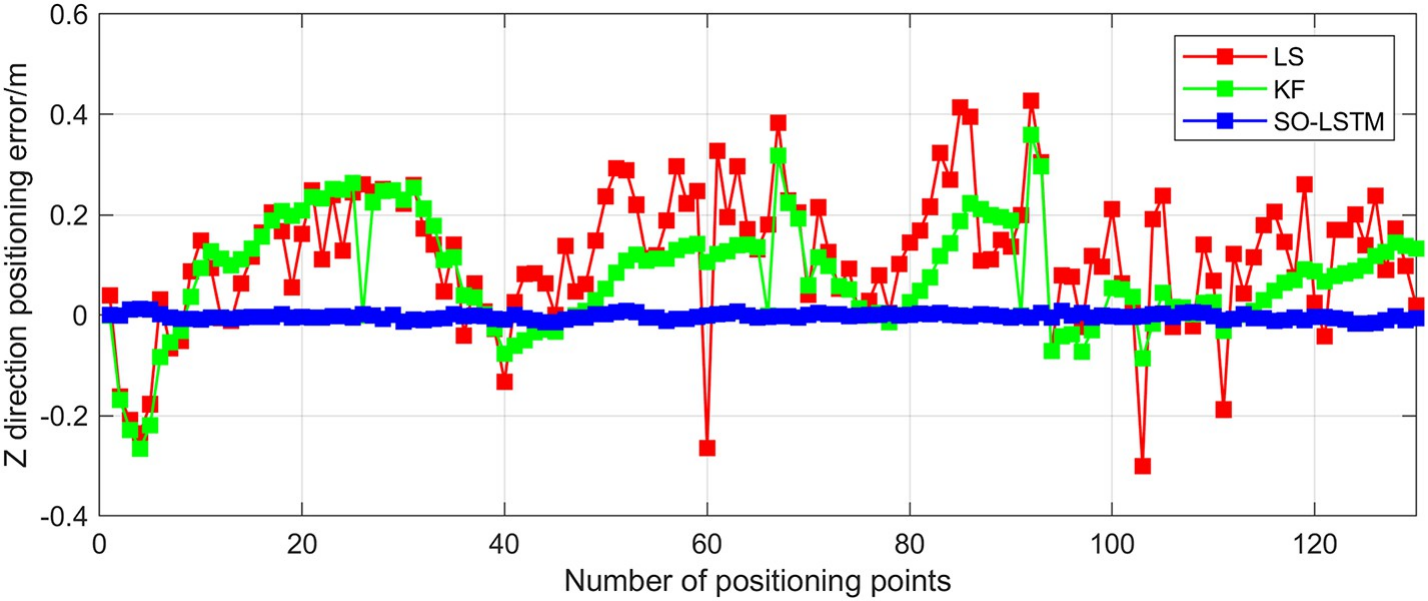
Experiment 1: Z-axis positioning estimation error.

This research conducted an analysis of the positional estimation errors of the three localization methods along the X, Y, and Z axes. Figs 11–13 serve to visually represent the positional estimation errors along these specific coordinate axes. Table 4 provides the RMSE values for the X, Y, and Z coordinate axes associated with the three positioning methods. The findings reveal that the SO-LSTM method demonstrates slightly improved position estimation errors in the X and Y axis components compared to the LS and KF methods. Furthermore, in the Z axis component, the SO-LSTM method significantly surpasses the LS and KF methods in terms of performance.

**Table 4:** RMSE for all three axes in experiment 1 with three positioning methods.

	LS	KF	SO-LSTM
RMSE of the X-axis (m)	0.0547	0.0441	0.0269
RMSE of the Y-axis (m)	0.0741	0.0544	0.0433
RMSE of the Z-axis (m)	0.1772	0.1339	0.0062

### 5.3. Experiment 2

**Fig 14:**
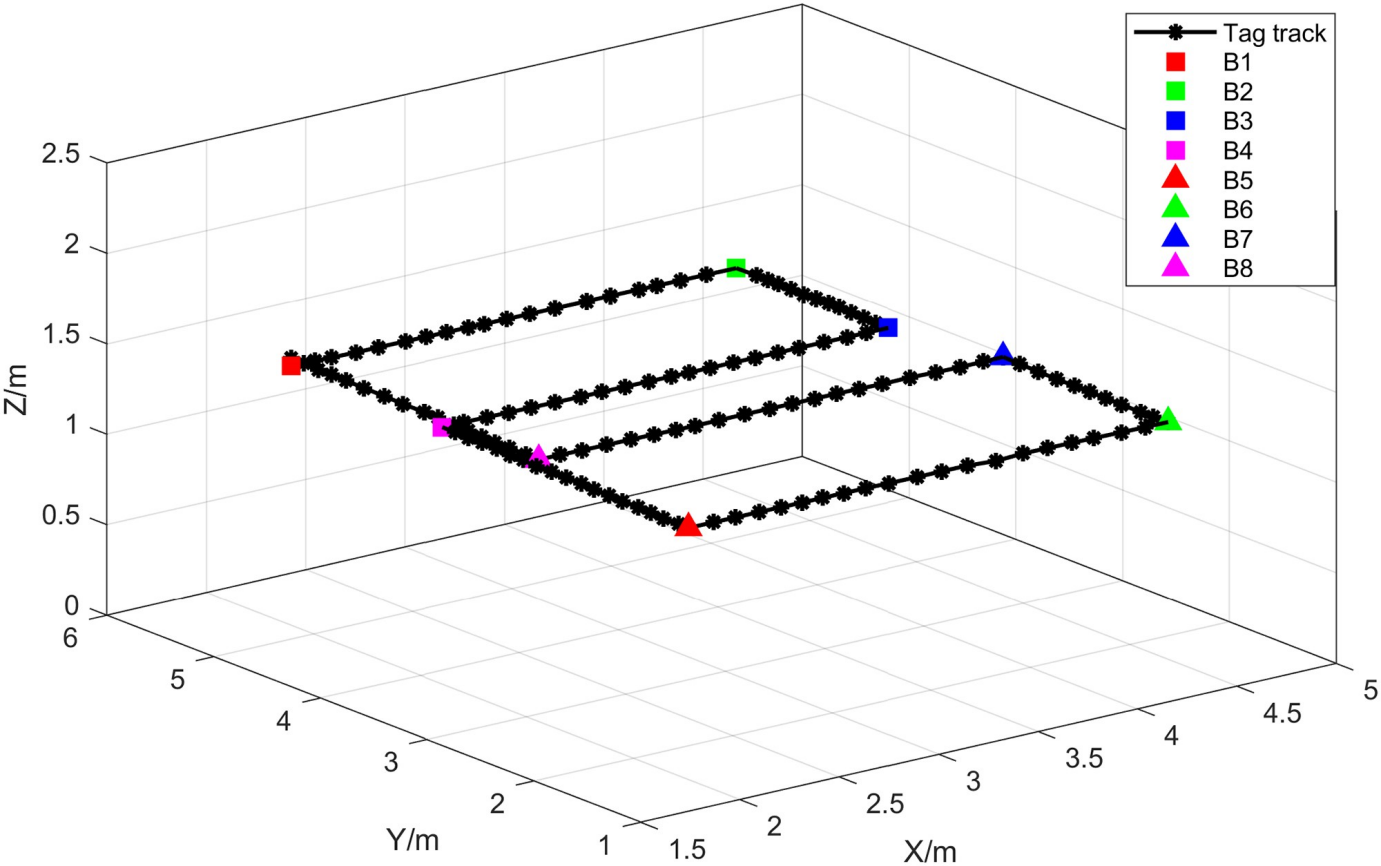
Experimental trajectory of crane hook movement in experiment 2.

In the second set of experiments, the positions of the Anchors and other conditions remained unchanged, but the movement path of the crane hook was modified. The initial position of the Tag was set at B1 (2.726, 5.716, 1.248), with turning points at B2 (4.484, 5.659, 1.247), B3 (4.476, 4.219, 1.249), B4 (2.745, 4.265, 1.247), B5 (2.787, 1.879, 1.247), B6 (4.594, 1.817, 1.249), B7 (4.603, 3.381, 1.248), and B8 (2.706, 3.433, 1.248). The movement trajectory of the crane hook followed the sequence B1 → B2 → B3 → B4 → B5 → B6 → B7 → B8 → B1. Distance measurements between the Tag and the Anchors were collected during the crane operation. Fig 14 illustrates the movement trajectory of the crane hook in the second experiment.

**Fig 15:**
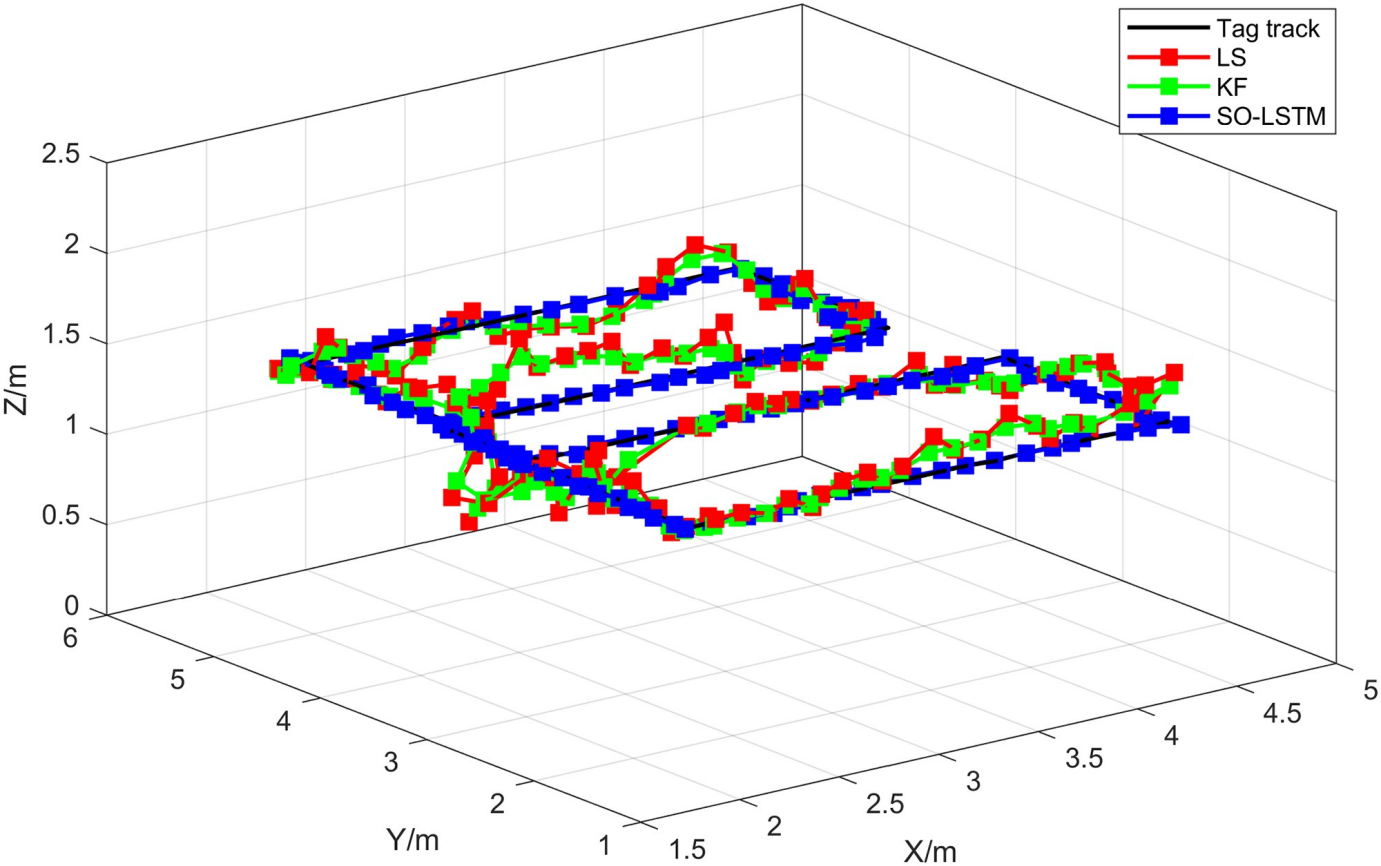
Localization trajectory for experiment 2.

In this set of experiments, the Snake Optimizer algorithm, obtained from Experiment 1, was employed to determine the LSTM network hyperparameters for the position estimation process of the SO-LSTM localization method. Fig 15 illustrates the true trajectory of the target and the estimated trajectories obtained by the LS, KF, and SO-LSTM localization methods in the second set of experiments. As shown in Table 5. The RMSE and MPE were calculated for each localization method. The LS method yielded an RMSE of 0.1609 meters and an MPE of 0.2868 meters. The KF method achieved an RMSE of 0.1403 meters and an MPE of 0.2376 meters. The SO-LSTM localization method demonstrated superior performance with an RMSE of 0.0589 meters and an MPE of 0.1125 meters. These results indicate that the SO-LSTM localization method surpasses the LS and KF methods in terms of position tracking accuracy.

**Table 5:** Statistical analysis of positioning errors for three localization methods in experiment 2.

	LS	KF	SO-LSTM
RMSE (m)	0.1609	0.1403	0.0589
MPE (m)	0.2868	0.2376	0.1125

**Fig 16:**
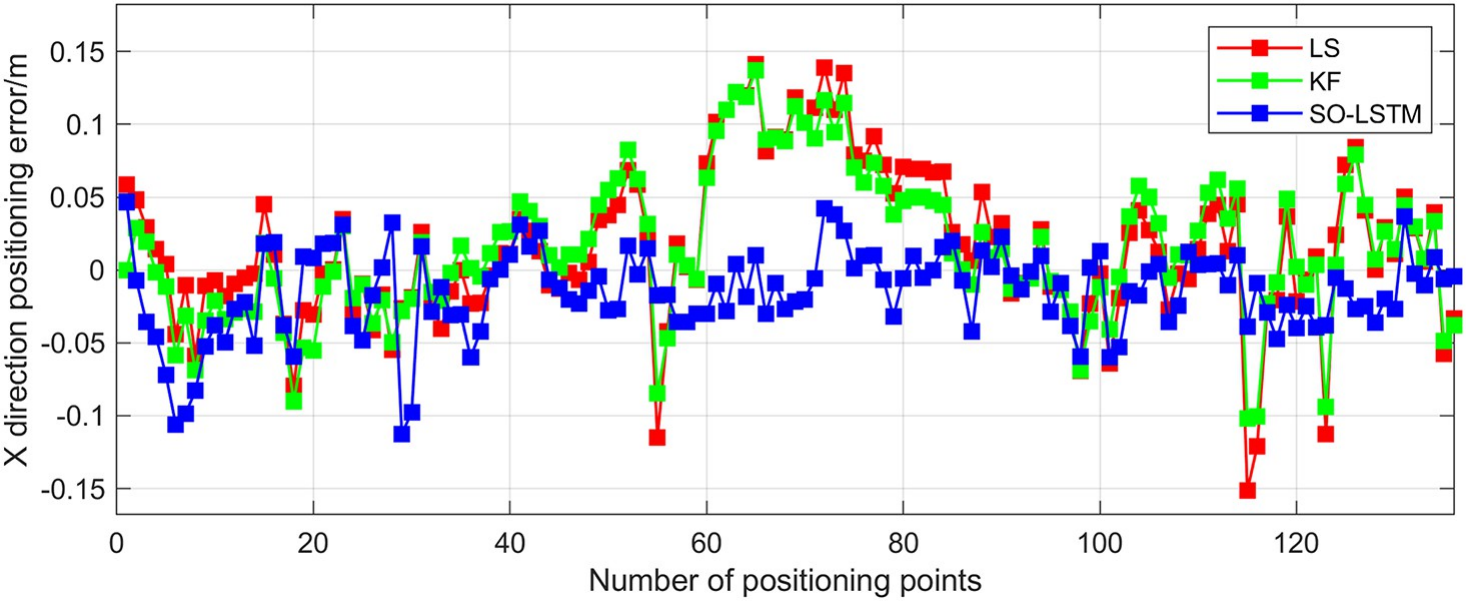
Experiment 2: X-axis positioning estimation error.

**Fig 17:**
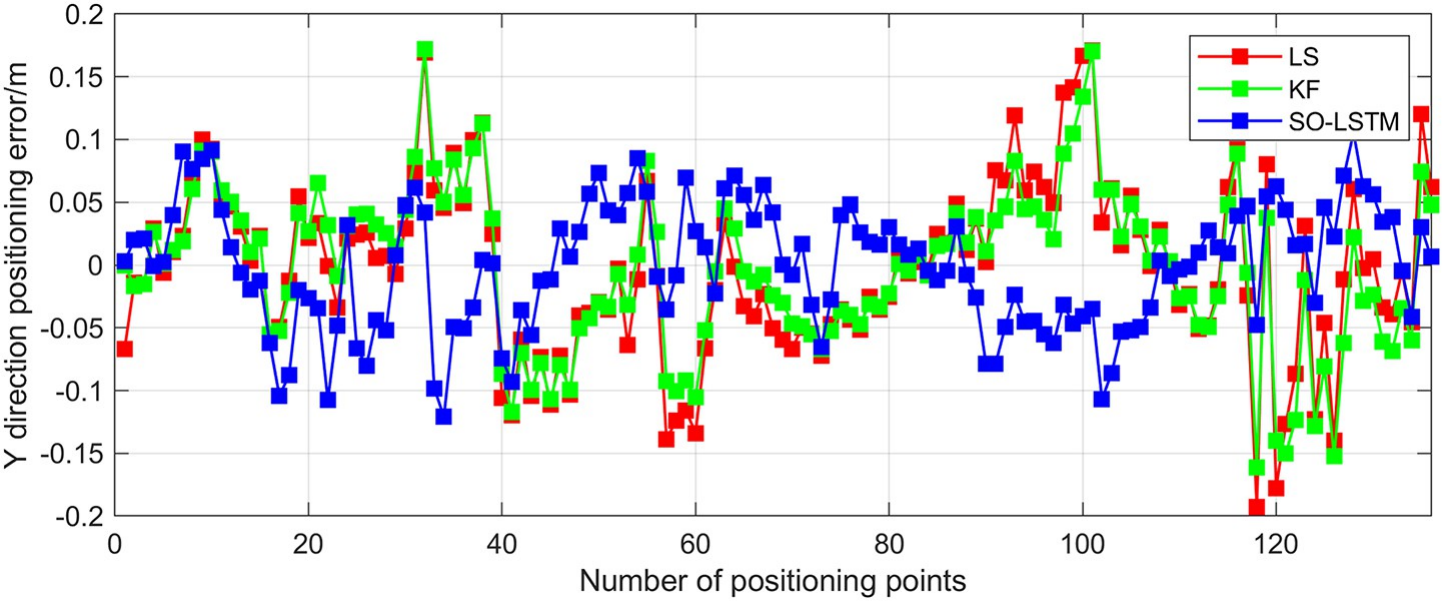
Experiment 2: Y-axis positioning estimation error.

**Fig 18:**
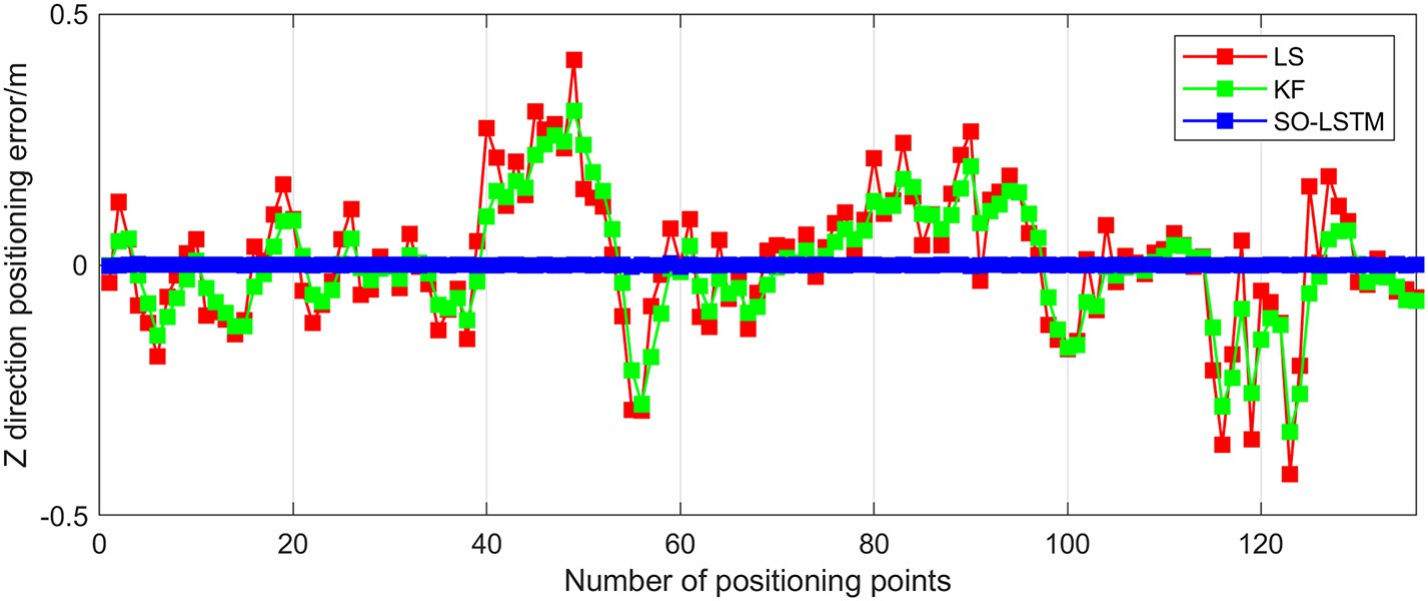
Experiment 2: Z-axis positioning estimation error.

This study undertakes an analysis of the positioning errors incurred by three distinct estimation methods concerning the X, Y, and Z coordinate axes. Notably, the graphical representations in Figs 16–18 provide a comprehensive visualization of the positioning estimation errors along these specific axes. Table 6 provides the RMSE values for the three localization methods in the X, Y, and Z coordinate axes. Notably, the SO-LSTM localization method demonstrates superior positioning accuracy in all three coordinates (X, Y, and Z) when compared to the LS and KF methods.

**Table 6:** RMSE for all three axes in experiment 2 with three positioning methods.

	LS	KF	SO-LSTM
RMSE of the X-axis (m)	0.0550	0.0511	0.0329
RMSE of the Y-axis (m)	0.0693	0.0639	0.0489
RMSE of the Z-axis (m)	0.1344	0.1140	0.0008

### 5.4. Results and analysis

**Fig 19:**
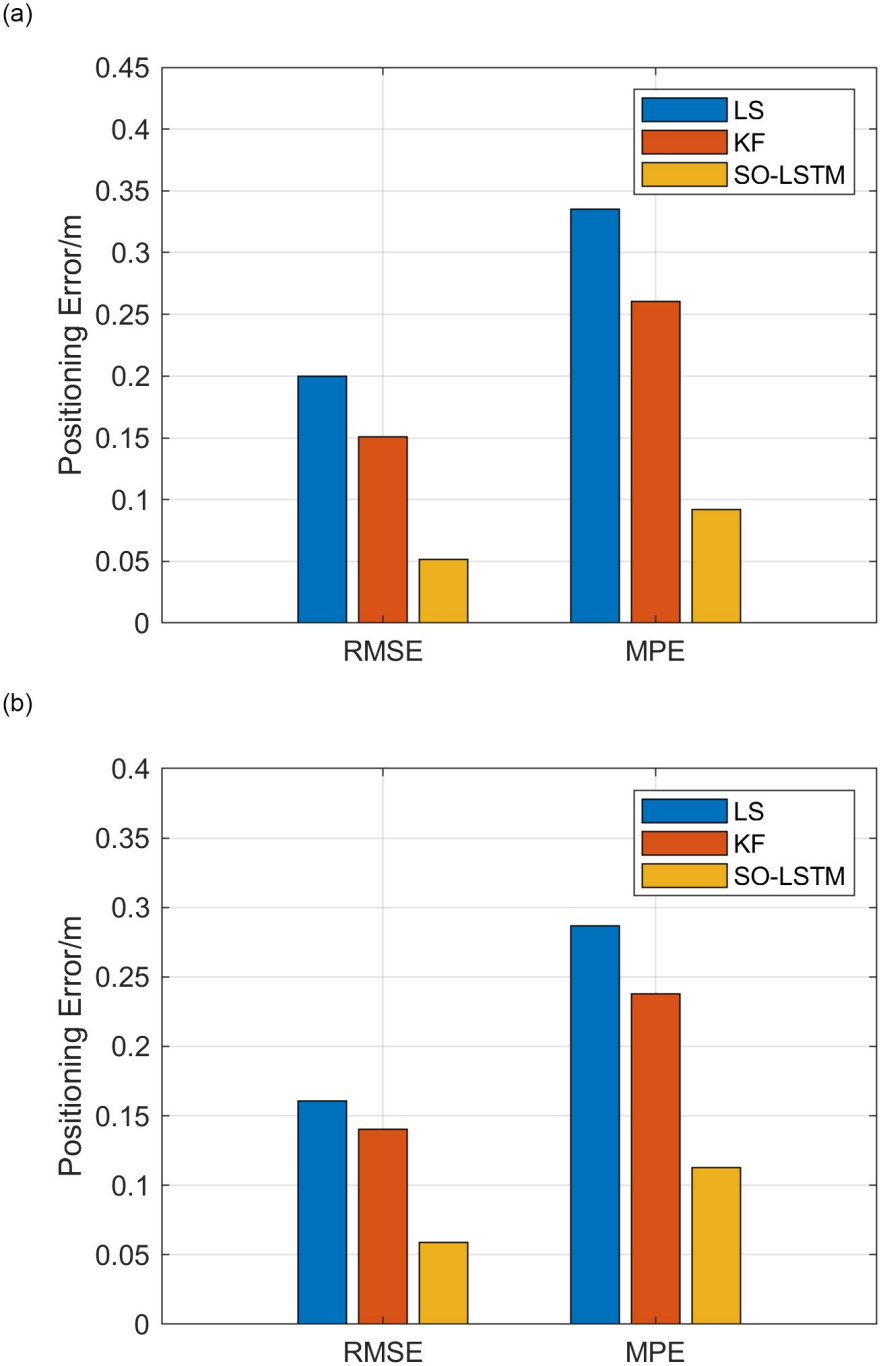
Analysis of position estimation trajectory errors (a) experiment 1 error histogram (b) experiment 2 error histogram.

Fig 19(a) and 19(b) in the experiment depict the histograms of RMSE and MPE for the three positioning estimation methods employed in Experiment 1 and Experiment 2, respectively. The histograms clearly illustrate that the SO-LSTM positioning method consistently exhibits smaller values of RMSE and MPE in comparison to the LS and KF methods.

Table 7 presents the percentage improvement in positioning accuracy achieved by the SO-LSTM method when compared to the LS and KF methods. The results indicate that in Experiment 1, the SO-LSTM positioning method achieved a significant reduction of 74.33% in RMSE and 72.65% in MPE compared to the LS method. Similarly, compared to the KF method, the SO-LSTM positioning method achieved a reduction of 66.02% in RMSE and 64.75% in MPE. In Experiment 2, the SO-LSTM positioning method demonstrated a reduction of 63.39% in RMSE and 60.77% in MPE compared to the LS method. Furthermore, when compared to the KF method, the SO-LSTM positioning method achieved a reduction of 58.01% in RMSE and 52.65% in MPE.

**Table 7:** Percentage improvement in SO-LSTM positioning accuracy.

		LS positioning error (m)	KF positioning error (m)	SO-LSTM positioning error (m)	Percentage improvement in accuracy of SO-LSTM compared to LS.	Percentage improvement in accuracy of SO-LSTM compared to KF.
Experiment 1	MPE	0.3354	0.2602	0.0917	72.65%	64.75%
	RMSE	0.1999	0.1510	0.0513	74.33%	66.02%
Experiment 2	MPE	0.2868	0.2376	0.1125	60.77%	52.65%
	RMSE	0.1609	0.1403	0.0589	63.39%	58.01%

Through the analysis of the positioning errors in Experiment 1 and Experiment 2, it can be concluded that the proposed SO-LSTM positioning method exhibits a significant enhancement in the accuracy of the UWB positioning system compared to the LS and KF methods. This finding highlights the effectiveness of the SO-LSTM method in improving the precision of location estimation, particularly in unmanned crane systems. The results suggest that the SO-LSTM method offers a promising solution for achieving higher accuracy in position estimation.

## 6. Conclusions

This paper presents a novel UWB positioning methodology for unmanned crane systems, employing the SO-LSTM technique with the objective of enhancing the positioning accuracy of the crane’s hook. The proposed method leverages the TDMA and TWR techniques to establish a multi-base station and multi-tag UWB positioning system. Initially, the LSTM network’s hyperparameters, including the number of hidden nodes, learning rate, and iteration count, are optimized using the Snake Optimizer algorithm. Subsequently, the optimized LSTM network is trained using UWB ranging data to precisely determine the position of the crane’s hook.

Experimental findings substantiate that the proposed approach outperforms the conventional LS and KF methods in terms of achieving higher accuracy in position estimation. The proposed method effectively addresses the limitations associated with traditional crane positioning techniques, which encounter challenges in accurately determining the position of swinging loads. In the future, the application of the SO-LSTM-based UWB positioning method in real-world crane operation scenarios will be conducted to further assess its practical viability and suitability.

## Supporting information

S1 File In the S1 File, there are six tables included. The tables "Experiment 1 Raw Data" and "Experiment 2 Raw Data" contain the raw data for Experiment 1 and Experiment 2, respectively. The tables "Experiment 1: Three Methods for Localization Results" and "Experiment 2: Three Methods for Localization Results" display the localization results of the three methods in Experiment 1 and Experiment 2. The tables "Experiment 1 Positioning Error" and "Experiment 2 Positioning Error" provide the positioning estimation errors for Experiment 1 and Experiment 2.
(XLSX)
